# Higher vs. lower positive end-expiratory pressure during one-lung ventilation for thoracic surgery: a systematic review and meta-analysis

**DOI:** 10.3389/fsurg.2026.1838287

**Published:** 2026-05-11

**Authors:** Xinrui Yin, Shijia Du

**Affiliations:** 1 Department of Anesthesiology, Aerospace Center Hospital, Beijing, China; 2 Department of VIP Dental Service, Peking University Stomatological Hospital, Beijing, China

**Keywords:** intraoperative hypotension, meta-analysis, one-Lung ventilation, positive end-expiratory pressure, postoperative pulmonary complications

## Abstract

**Background:**

Higher fixed positive end-expiratory pressure (PEEP) during one-lung ventilation (OLV) may improve intraoperative oxygenation but could compromise haemodynamic stability. We performed a systematic review and meta-analysis to evaluate the effects of higher vs. lower fixed PEEP strategies on intraoperative hypotension and postoperative pulmonary complications (PPCs) in patients undergoing thoracic surgery.

**Methods:**

We searched MEDLINE, Embase, and CENTRAL from inception to 7 March 2026, supplemented by trial registries. Parallel-group randomised controlled trials (RCTs) comparing higher vs. lower fixed PEEP during OLV were eligible. Co-primary outcomes were intraoperative hypotension and PPCs. Pooled risk ratios (RRs) and mean differences (MDs) were estimated using random-effects models with restricted maximum likelihood (REML) estimation. Certainty of evidence was assessed using GRADE. This review was registered in PROSPERO (CRD420261329237).

**Results:**

Eight RCTs (2,747 patients) were included. Higher PEEP was associated with a significantly increased risk of intraoperative hypotension (2 studies; *n* = 2,086; RR: 2.16, 95% CI: 1.29–3.63; *p* = 0.003; *I*^2^ = 61%; moderate certainty). Higher PEEP did not significantly reduce the risk of PPCs (2 studies; *n* = 2,227; RR: 0.95, 95% CI: 0.88–1.02; *p* = 0.179; *I*^2^ = 0%; moderate certainty). New-onset arrhythmia was more frequent with higher PEEP (RR: 2.56, 95% CI: 1.76–3.71), while rescue hypoxaemia interventions were less frequent (RR: 0.37, 95% CI: 0.25–0.56). Pooled vasopressor use did not differ significantly between groups (RR: 1.05, 95% CI: 0.97–1.13). Intraoperative PaO_2_ was directionally higher with higher PEEP but the estimate was statistically inconclusive owing to substantial heterogeneity (*I*^2^ = 94.2%).

**Conclusions:**

Higher fixed PEEP during OLV significantly increases intraoperative hypotension risk without reducing PPCs. The haemodynamic cost of this strategy is not offset by measurable clinical benefit. Routine application of higher fixed PEEP during OLV should be approached with caution, and future research should evaluate individualised PEEP titration strategies.

**Systematic Review Registration:**

https://www.crd.york.ac.uk/prospero/display_record.php?ID=CRD420261329237, identifier CRD420261329237.

## 1 Introduction

One-lung ventilation (OLV) is a fundamental component of thoracic anaesthesia, enabling surgical exposure of the operative hemithorax while maintaining gas exchange through the dependent lung. However, OLV imposes substantial physiological stress. The non-ventilated lung is prone to absorption atelectasis, while gas exchange depends on hypoxic pulmonary vasoconstriction to limit shunt. At the same time, the dependent ventilated lung must accommodate the full tidal ventilation, increasing susceptibility to overdistension and cyclic alveolar collapse (1, 2). Postoperative pulmonary complications (PPCs) remain among the most common and clinically important adverse outcomes after thoracic surgery, with reported incidences ranging from approximately 15%–30% depending on the procedure and patient risk profile, and are associated with increased postoperative morbidity, prolonged hospital stay, and increased healthcare utilisation (3, 4). Optimising intraoperative ventilation during OLV is therefore a priority in perioperative care.

Positive end-expiratory pressure (PEEP) is a central modifiable component of the OLV ventilation strategy. By maintaining alveolar patency at end-expiration, PEEP may reduce atelectasis and improve oxygenation in the dependent ventilated lung (5). However, the optimal PEEP level during OLV remains uncertain. Higher PEEP may improve intraoperative oxygenation and respiratory mechanics, but may also compromise haemodynamic stability by reducing venous return and increasing right ventricular afterload (6). Several small single-centre randomised controlled trials (RCTs) have compared higher vs. lower fixed PEEP strategies during OLV, often suggesting directionally favourable effects on oxygenation and dynamic compliance with higher PEEP, but these studies were individually underpowered to assess clinically important postoperative outcomes (7–12). More recently, the PROTHOR trial, a large multicentre international phase 3 RCT, provided the most definitive evidence to date and suggested that higher PEEP with recruitment manoeuvres may not reduce PPCs compared with lower PEEP, while increasing intraoperative haemodynamic adverse events (13). These findings challenge the assumption that higher PEEP uniformly benefits patients undergoing OLV and raise the possibility that its haemodynamic cost may be consistent across different surgical settings and patient risk profiles.

Against this background, a systematic synthesis of all available randomised evidence is warranted. Such a synthesis is needed to characterise the trade-off between the potential physiological benefits of higher fixed PEEP during OLV, such as improved intraoperative oxygenation, and its clinical haemodynamic risks, and to contextualise the PROTHOR findings within the broader evidence base. We therefore conducted a systematic review and meta-analysis of parallel-group RCTs comparing higher vs. lower fixed PEEP strategies during OLV for thoracic surgery to evaluate their effects on intraoperative haemodynamic safety and postoperative pulmonary complications, with co-primary outcomes of intraoperative hypotension and PPCs. This review was prospectively registered in PROSPERO (CRD420261329237) and conducted in accordance with the PRISMA 2020 statement (14).

## 2 Methods

### 2.1 Study design and registration

This systematic review and meta-analysis was designed *a priori* and prospectively registered in the International Prospective Register of Systematic Reviews (PROSPERO; registration number CRD420261329237; registered on 1 March 2026). The conduct and reporting of this review followed the Preferred Reporting Items for Systematic Reviews and Meta-Analyses (PRISMA) 2020 statement. The PRISMA 2020 checklist is provided in the Supplementary Material. No material deviations from the registered protocol occurred (14).

### 2.2 Eligibility criteria

Eligibility criteria were prespecified using the Population–Intervention–Comparator–Outcomes–Study design (PICOS) framework, with outcome definitions detailed separately below.

#### 2.2.1 Population

We included adult patients (aged ≥18 years) undergoing elective thoracic surgery requiring intraoperative one-lung ventilation (OLV) under general anaesthesia with mechanical ventilation. Eligible procedures included lung resections (e.g., lobectomy, segmentectomy, wedge resection, pneumonectomy), oesophagectomy, and other thoracic operations requiring OLV. Both video-assisted thoracoscopic surgery (VATS) and open thoracotomy were eligible. No restrictions were applied regarding sex, baseline comorbidity profile, or American Society of Anesthesiologists (ASA) physical status.

#### 2.2.2 Intervention

We included trials comparing a higher positive end-expiratory pressure (PEEP) strategy during OLV with a lower PEEP strategy. The higher PEEP strategy was defined as the arm assigned to the higher fixed PEEP level in each trial, generally above the comparator arm and most commonly in the range of 8–10 cmH_2_O, with or without a recruitment manoeuvre (RM). Details of the PEEP level and RM protocol (if applied) had to be extractable from the report.

#### 2.2.3 Comparator

The comparator was the lower fixed PEEP strategy during OLV (typically ≤5 cmH_2_O), without routine RM or with a less intensive or discretionary RM approach, as defined by each trial.

#### 2.2.4 Study design

We included parallel-group randomised controlled trials (RCTs). We imposed no restrictions on publication year, language, or country.

#### 2.2.5 Exclusion criteria

We excluded studies that: (i) compared two active PEEP titration approaches (e.g., electrical impedance tomography-guided vs. compliance-guided titration) without a fixed low-PEEP control arm; (ii) did not report PEEP levels or did not provide sufficient information to extract the PEEP strategy; (iii) were quasi-randomised or non-randomised comparative studies (these may be summarised narratively but were not eligible for the primary quantitative synthesis); or (iv) enrolled paediatric participants (<18 years) or populations in which OLV was not the primary intraoperative ventilation strategy.

### 2.3 Outcomes

Outcomes were prespecified and prioritised as co-primary and key secondary outcomes. Outcome definitions were extracted verbatim from each trial report where available.

#### 2.3.1 Co-primary outcomes

##### 2.3.1.1 Intraoperative hypotension

The primary hypotension endpoint was any intraoperative hypotension as defined by each trial (e.g., absolute or relative thresholds, duration criteria, or composite definitions); the trial-specific definition was recorded verbatim. To capture clinically meaningful haemodynamic compromise in a standardised manner across studies, we prespecified a second hypotension endpoint: clinically significant hypotension, defined as hypotension requiring vasoactive medication and/or vasopressor administration according to the trial protocol or reported rescue treatment. Because thresholds and indications for fluid bolus administration varied substantially across trials, fluid bolus requirement alone was not used to define clinically significant hypotension. It is acknowledged that trial-specific hypotension definitions varied substantially across included studies; this heterogeneity in outcome definition is addressed in the Results and Limitations sections. For both hypotension endpoints, the effect measure was the risk ratio (RR).

##### 2.3.1.2 Postoperative pulmonary complications (PPCs)

PPCs were defined as a composite outcome assessed during the in-hospital period or within the follow-up window reported by each trial. We prioritised the composite PPC definition as reported by each trial and recorded the definition verbatim. If a trial did not report an explicit PPC composite but reported one or more prespecified PPC components, we constructed a derived composite PPC outcome *a priori* by counting patients with any of the reported components (e.g., pneumonia, atelectasis, acute respiratory failure/ARDS, reintubation, prolonged ventilation) and included this derived composite in the primary analysis to avoid unnecessary exclusion of eligible trials. To assess robustness, we planned a sensitivity analysis restricted to trials reporting an explicit composite PPC outcome. The effect measure for PPCs was RR.

#### 2.3.2 Key secondary outcomes

Secondary outcomes included: (1) vasopressor use during surgery (any use; RR). When trials reported dose or cumulative exposure, these were synthesised as mean difference (MD) only when the same drug, comparable units, and compatible exposure windows were reported by at least two trials; otherwise results were summarised narratively. (2) New-onset intraoperative arrhythmia (RR). (3) Intraoperative oxygenation, assessed preferentially using the PaO_2_/FiO_2_ ratio where available, or PaO_2_ when this was the most consistently extractable metric across trials, at prespecified timepoints after initiation of OLV (preferentially at approximately 30 min and 60 min when available; MD). (4) Rescue interventions for hypoxaemia during OLV (RR), as defined by each trial (e.g., increased FiO_2_, CPAP to the non-ventilated lung, intermittent two-lung ventilation, or other protocolised measures). (5) Individual PPC components were also analysed separately when available, including pneumonia, atelectasis, acute respiratory failure/ARDS, reintubation, and prolonged ventilation (RR for each). Additional postoperative outcomes included postoperative respiratory support (RR), ICU admission (RR), ICU length of stay (MD), hospital length of stay (MD), and 30-day mortality (RR) where available. Outcome-specific denominators were used when trials reported analysable populations that differed across endpoints.

### 2.4 Search strategy and information sources

We searched MEDLINE (via PubMed), Embase (via Embase.com), and the Cochrane Central Register of Controlled Trials (CENTRAL) from inception to 7 March 2026. We additionally searched ClinicalTrials.gov and the World Health Organization International Clinical Trials Registry Platform (WHO ICTRP) up to 7 March 2026 to identify ongoing or unpublished trials. Reference lists of all included studies and relevant systematic reviews were screened manually to identify additional eligible records.

The PubMed search strategy was prespecified *a priori* and combined controlled vocabulary and free-text terms for one-lung ventilation, positive end-expiratory pressure/recruitment manoeuvres, and thoracic surgery, together with a randomised trial filter. No restrictions were applied regarding language, publication year, or geographic location. The full PubMed search strategy is provided in Supplementary Appendix 1, and equivalent strategies were adapted for Embase and CENTRAL. Any minor refinements made during search execution to improve precision (e.g., adjustment of free-text trial filters) did not alter the underlying conceptual framework of the search. The surgical component of the search was built around the MeSH term “thoracic surgery” supplemented by procedure-specific free-text terms (lobectomy, pneumonectomy, esophagectomy/oesophagectomy, VATS, and “video-assisted thorac*”), which were intended to capture both open and minimally invasive thoracic approaches; “thoracotomy” was not included as a standalone free-text term, as it is subsumed within the broader MeSH heading and the procedure-specific terms employed. The final search was run on 7 March 2026.

All records were exported to EndNote for de-duplication prior to screening. Multiple reports of the same trial were collated and treated as a single study.

### 2.5 Study selection and data extraction

After de-duplication, titles and abstracts were screened independently by two reviewers against the prespecified eligibility criteria. Full texts were obtained for all records deemed potentially eligible, and eligibility was assessed independently by the same two reviewers. Disagreements at any stage were resolved through discussion and consensus between the two reviewers. The study selection process was documented using a PRISMA 2020 flow diagram, including reasons for exclusion at the full-text stage.

Data extraction was performed independently by two reviewers using a standardised, pilot-tested data extraction form. Extracted information included: trial identifiers (first author, year, country, trial registration), study design characteristics, sample size, funding and conflicts of interest; participant baseline characteristics (e.g., age, sex, BMI, ASA class, major comorbidities, type and approach of surgery); intraoperative ventilation and co-intervention details (PEEP levels in each group, tidal volume and whether indexed to predicted body weight, FiO_2_, ventilation mode, duration of OLV, and the RM protocol including type, pressure, duration, and frequency); and outcome data for all prespecified endpoints (definitions and event counts for dichotomous outcomes; means/standard deviations and timepoints for continuous outcomes).

When outcome data were incomplete, inconsistent, or reported only in figures, we sought to obtain the required information from Supplementary Materials, trial registry records, or by contacting study authors where feasible. When continuous outcomes were reported as medians with interquartile ranges and/or ranges, we converted these to means and standard deviations using established methods when sufficient summary statistics were available (15, 16). If conversion was not feasible, the outcome was not quantitatively pooled and was summarised narratively. For trials reported in multiple publications, all reports were collated, and the most complete dataset was used; discrepancies were resolved by cross-checking reports and prioritising prespecified outcomes and the longest available follow-up window consistent with the trial protocol.

Unit-of-analysis and multi-arm trials. The unit of analysis was the individual participant. For multi-arm trials with more than two eligible PEEP groups, we planned to avoid double-counting by combining intervention groups when clinically appropriate; if combining was not appropriate, we would split the shared comparator group evenly across relevant comparisons in accordance with Cochrane guidance (17).

### 2.6 Risk of bias and data synthesis

#### 2.6.1 Risk of bias assessment

Risk of bias for each included trial was assessed independently by two reviewers using the Cochrane Risk of Bias tool for randomised trials (RoB 2) (18). The following five domains were evaluated: (1) bias arising from the randomisation process; (2) bias due to deviations from intended interventions; (3) bias due to missing outcome data; (4) bias in measurement of the outcome; and (5) bias in selection of the reported result. Each domain was judged as low risk, some concerns, or high risk of bias, leading to an overall risk-of-bias judgement for each trial. Disagreements were resolved by discussion and consensus between the two reviewers. Risk-of-bias assessments were summarised in both tabular and graphical formats.

#### 2.6.2 Effect measures

For dichotomous outcomes, treatment effects were summarised as risk ratios (RRs) with 95% confidence intervals (CIs), calculated from 2 × 2 tables (events/total) whenever possible. Odds ratios (ORs) were explored in prespecified sensitivity analyses. For continuous outcomes, effects were summarised as mean differences (MDs) with 95% CIs when outcomes were reported using the same scale and units; standardised mean differences (SMDs) would be used if different scales were used to measure the same construct.

#### 2.6.3 Meta-analysis model and heterogeneity

The primary meta-analyses were conducted using a random-effects model to account for anticipated clinical and methodological heterogeneity across trials. Between-study variance (*τ*²) was estimated using restricted maximum likelihood (REML). Statistical heterogeneity was quantified using the *I*^2^ statistic and *τ*², and assessed using the Cochran Q test. Predefined thresholds for interpretation of *I*^2^ were used cautiously, with *I*^2^ > 50% considered suggestive of substantial heterogeneity, prompting exploration through prespecified subgroup analyses and narrative assessment.

#### 2.6.4 Prespecified subgroup analyses

Where sufficient data were available, we conducted subgroup analyses to explore potential sources of heterogeneity according to: (1) type of surgery (lung resection vs. oesophagectomy vs. mixed populations); (2) surgical approach (predominantly VATS vs. predominantly open thoracotomy); (3) level of higher PEEP (≥10 cmH_2_O vs. 8–9 cmH_2_O); (4) recruitment manoeuvre strategy (standardised/protocolised RM vs. no RM vs. variable/discretionary RM); and (5) trial size (≥200 vs. <200 participants). Subgroup effects were interpreted cautiously, considering the number of trials and biological/clinical plausibility.

#### 2.6.5 Prespecified sensitivity analyses

We conducted sensitivity analyses to assess robustness of the primary findings by: (1) excluding trials at overall high risk of bias; (2) excluding the PROTHOR trial to evaluate its potential influence on pooled estimates given its large sample size; (3) using alternative pooling assumptions (fixed-effect inverse-variance model; DerSimonian–Laird random-effects model); (4) using ORs instead of RRs for dichotomous outcomes; and (5) restricting analyses to trials with an absolute between-group PEEP difference ≥5 cmH_2_O.

#### 2.6.6 Publication bias and small-study effects

Assessment of publication bias and small-study effects using funnel plots and Egger's regression test was planned only when at least 10 trials contributed to a given meta-analysis. When fewer than 10 trials were available, we did not formally test for publication bias and stated this limitation explicitly.

#### 2.6.7 Certainty of evidence

The certainty of evidence for the co-primary outcomes and key secondary outcomes was assessed using the GRADE framework (19), considering risk of bias, inconsistency, indirectness, imprecision, and publication bias. A Summary of Findings table was prepared to present pooled estimates and the certainty rating for the most clinically important outcomes.

#### 2.6.8 Software

All meta-analyses were performed using R (packages meta and metafor) and/or RevMan 5 (20, 21), with forest plots generated in R, RevMan, or Stata as appropriate. Where reported, we prioritised effect estimates based on the intention-to-treat principle.

## 3 Results

### 3.1 Study selection

**Figure 1:**
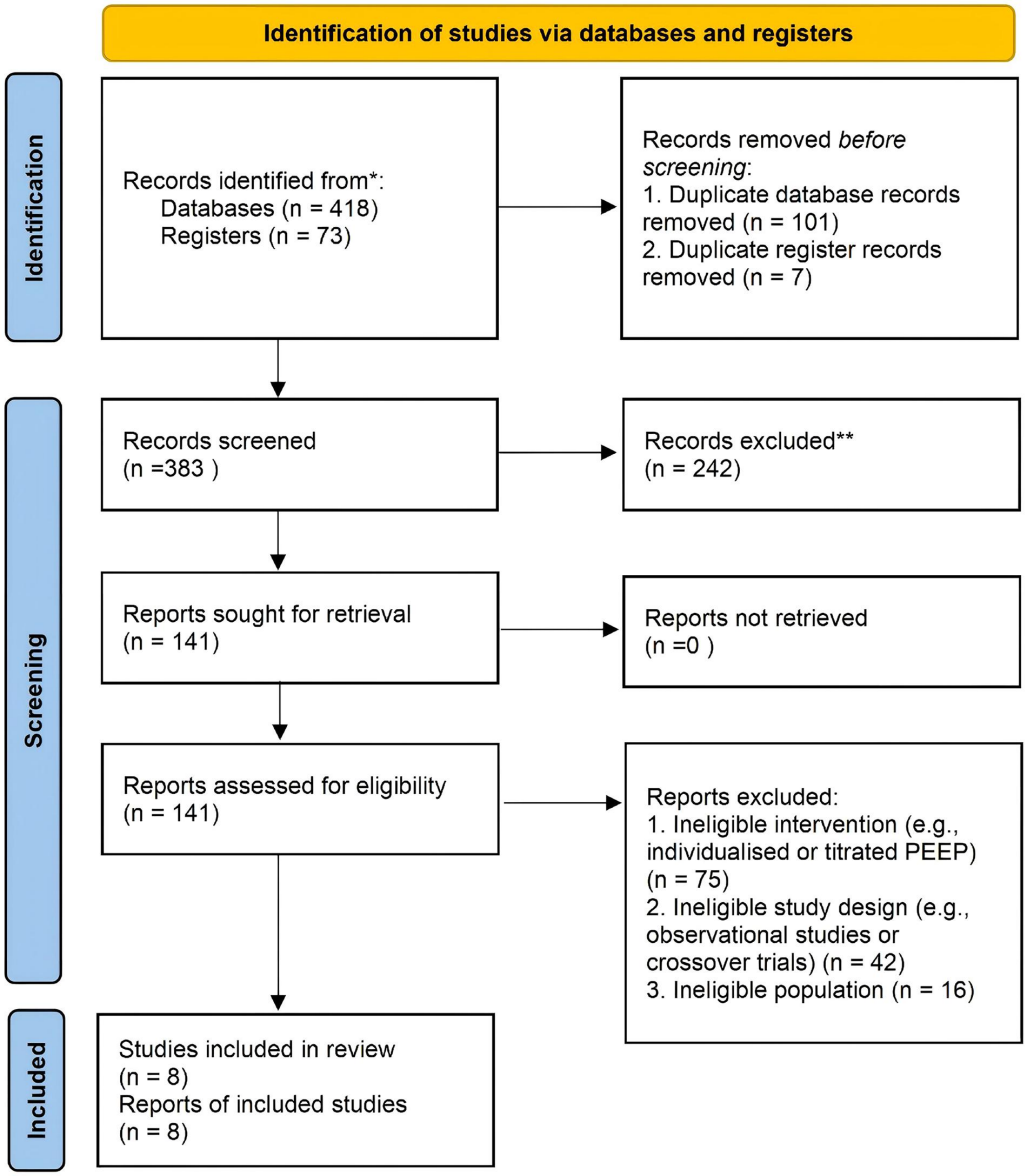
PRISMA 2020 flow diagram of study selection.

A total of 418 records were identified through database searching (MEDLINE, Embase, and CENTRAL), and 73 additional records were identified through trial registers. After removal of 101 duplicate database records and 7 duplicate register records, the remaining 383 records (317 from databases and 66 from registers) underwent title and abstract screening. Of these, 242 records were excluded, and 141 reports were sought for full-text assessment. All reports sought for retrieval were successfully obtained for full-text assessment. After full-text review, 133 reports were excluded for the following reasons: ineligible intervention (e.g., individualised or titrated PEEP) (*n* = 75), ineligible study design (e.g., observational studies or crossover trials) (*n* = 42), and ineligible patient population (*n* = 16). Ultimately, 8 studies met the eligibility criteria and were included in the review. Of these, 7 studies were fully eligible for the primary review question, and 1 three-arm study contributed only the control and fixed-PEEP arms to the primary quantitative synthesis because the alveolar recruitment arm introduced an additional intervention. The study selection process is summarised in the PRISMA flow diagram (Figure 1).

Figure 1 legend (caption): PRISMA, Preferred Reporting Items for Systematic Reviews and Meta-Analyses. Records identified from MEDLINE, Embase, and CENTRAL were de-duplicated prior to screening. One included three-arm study (23) contributed only the control and fixed-PEEP arms to the primary quantitative synthesis; the alveolar recruitment arm was excluded from the primary analysis. Full-text exclusion reasons: ineligible intervention (e.g., individualised or titrated PEEP), *n* = 75; ineligible study design (e.g., observational studies or crossover trials), *n* = 42; ineligible population, *n* = 16.

### 3.2 Study characteristics

The review included 8 randomised controlled trials published between 2004 and 2026, comprising 1 large multicentre international phase 3 trial and 7 single-centre trials. The included studies were conducted in Europe and Asia, with thoracic surgery populations including patients undergoing lung resection, oesophagectomy, and other thoracic procedures requiring one-lung ventilation. Surgical approaches included both open thoracotomy and video-assisted thoracoscopic surgery (VATS). Characteristics of all included studies are presented in Table 1.

**Table 1:** Characteristics of included studies.

Study (Year)	Country/center Type	N (Analyzed)*	Surgery type	Surgical Approach	Higher PEEP (cmH_2_O)	Lower PEEP (cmH_2_O)	RM During OLV	Overall RoB 2
PROTHOR (2026)	28 countries/Multicentre	2,124	Mixed (lung resection, oesophagectomy)	Open/VATS	10	5	Yes	Low
Yoo et al. (9)	South Korea/Single-centre	137	Lung resection	VATS	9 or 6	3	No	Low
Kim et al. (24)	South Korea/Single-centre	48	Lung resection	VATS	6	0	No	Some concerns
Li et al. (10)	China/Single-centre	116	Oesophagectomy	Open	10 or 8	5 or 0	No	Some concerns
Siyahkoç et al. (22)	Turkey/Single-centre	40	Thoracic surgery	Open	10	5	No	Some concerns
Choi et al. (23)	South Korea/Single-centre	62	Anterior mediastinal lesions	VATS	8	0	No	Low
Leong et al. (7)	UK/Single-centre	42	Lung resection	Open	10 or 8	5 or 0	No	Some concerns
Valenza et al. (8)	Italy/Single-centre	46	Open-chest thoracic surgery	Open	10	0	No	Some concerns

Data are presented for the arms included in the primary quantitative synthesis. For multi-arm trials (7, 9, 10), *N* represents the total number of patients across all arms contributing to the meta-analysis. For (23), *N* = 62 represents only the fixed-PEEP and control arms; the alveolar recruitment arm was excluded from the primary analysis. Recruitment manoeuvres applied exclusively at the end of surgery or prior to extubation (9, 22) were not considered an intraoperative component of the PEEP strategy during one-lung ventilation and are therefore classified as absent in this column.

PEEP, positive end-expiratory pressure; OLV, one-lung ventilation; RM, recruitment manoeuvre during OLV; RoB 2, Risk of Bias 2 tool; VATS, video-assisted thoracoscopic surgery.

The largest study was the PROTHOR trial, which enrolled 2,200 randomised patients across 74 sites in 28 countries, whereas the remaining trials were substantially smaller and primarily focused on physiological intraoperative outcomes. Across studies, the higher PEEP strategy ranged from 6 to 10 cmH_2_O, while comparator groups used lower PEEP levels ranging from 0 to 5 cmH_2_O. Three studies used multi-arm fixed-PEEP designs, which were prespecified to be combined into clinically relevant lower- vs. higher-PEEP groups for the primary synthesis, as detailed in Table 1. One three-arm study comparing control, fixed PEEP, and alveolar recruitment plus PEEP contributed only the control vs. fixed PEEP comparison to the primary analysis.

Tidal volumes during one-lung ventilation varied across trials, ranging from approximately 4–10 mL/kg, although within individual trials the tidal volume strategy was generally kept constant across the PEEP comparison arms. Recruitment manoeuvres were incorporated in some studies, most notably in PROTHOR, where periodic lung recruitment was applied exclusively to the higher PEEP arm. In one trial (Siyahkoç, 2017) (22), an identical recruitment manoeuvre was applied to both PEEP groups and therefore did not represent a between-group difference. Outcome reporting was heterogeneous. The PROTHOR trial contributed the most clinically important postoperative and haemodynamic event data, including postoperative pulmonary complications (PPCs), intraoperative hypotension, arrhythmias, and rescue hypoxaemia interventions. In contrast, most smaller trials mainly reported physiological endpoints, such as PaO_2_, PaO_2_/FiO_2_, dynamic compliance, airway pressures, and shunt-related variables.

### 3.3 Risk of bias of included studies

**Figure 2:**
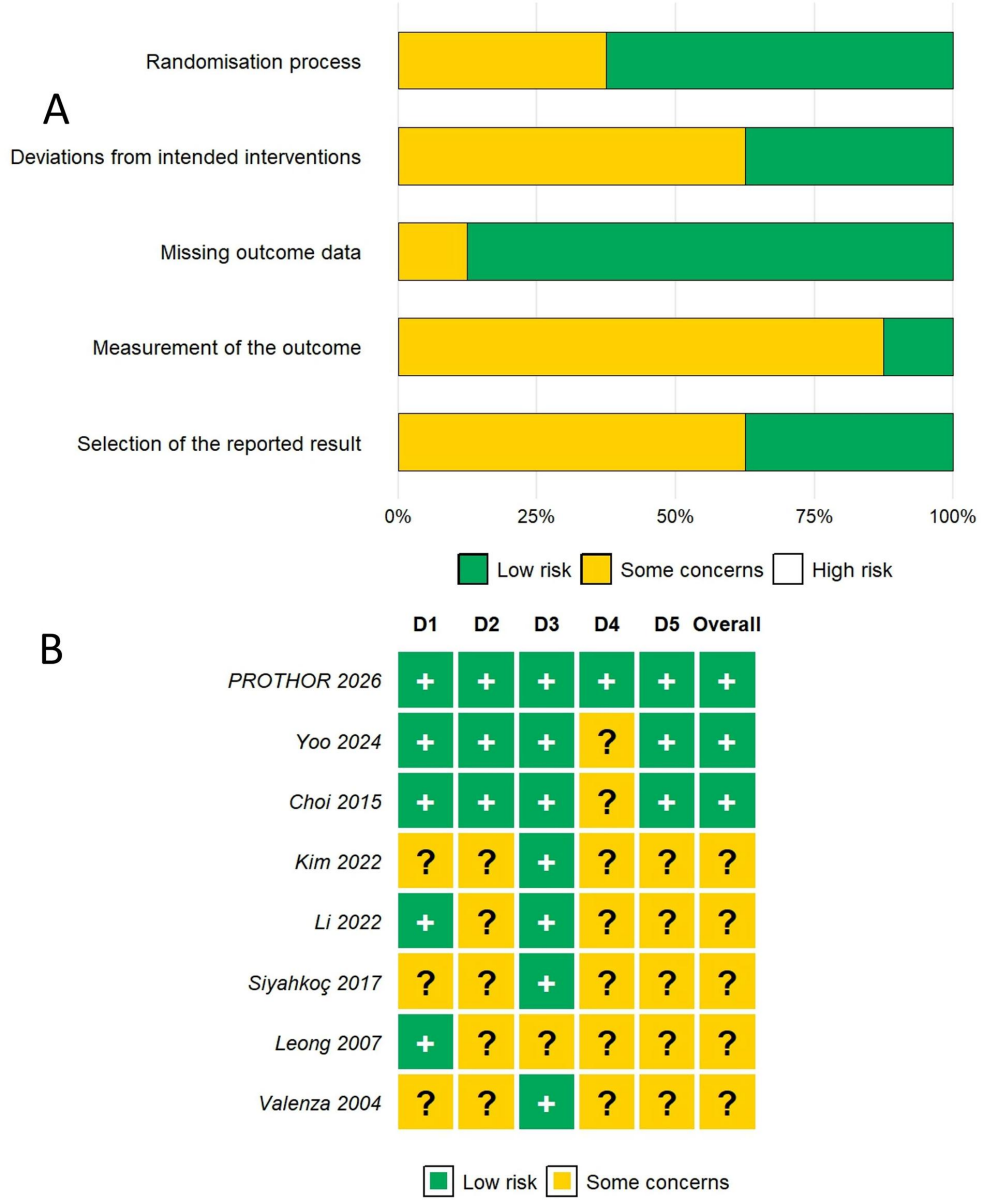
Risk of bias assessment of included studies. Risk of bias was assessed using the revised Cochrane risk-of-bias tool for randomised trials (RoB 2) across five domains. **(A)** Summary bar plot showing the proportion of included studies (*n* = 8) judged at low risk of bias or raising some concerns for each domain. No study was judged to be at high risk of bias. **(B)** Traffic light plot showing the domain-level and overall risk of bias judgement for each individual study. '+', low risk of bias; '?', some concerns. D1, randomisation process; D2, deviations from intended interventions; D3, missing outcome data; D4, measurement of the outcome; D5, selection of the reported result.

Overall, 3 studies were judged to be at low risk of bias, and 5 studies were judged to raise some concerns; no included study was judged to be at high risk of bias (Figures 2A,B). The trials judged at low risk of bias were the PROTHOR trial (13), Yoo et al. (9), and Choi et al. (23). Common reasons for assigning some concerns included limited reporting of allocation concealment, unclear blinding of intraoperative personnel or outcome assessors, and incomplete methodological detail in older or small single-centre trials.

Risk of bias was assessed using the revised Cochrane risk-of-bias tool for randomised trials (RoB 2) across five domains. (A) Summary bar plot showing the proportion of included studies (*n* = 8) judged at low risk of bias or raising some concerns for each domain. No study was judged to be at high risk of bias. (B) Traffic light plot showing the domain-level and overall risk of bias judgement for each individual study. “+”, low risk of bias; “?”, some concerns. D1, randomisation process; D2, deviations from intended interventions; D3, missing outcome data; D4, measurement of the outcome; D5, selection of the reported result.

The PROTHOR trial had the most robust methodological reporting, with central randomisation, masking of postoperative assessors, protocol registration, and modified intention-to-treat analysis. By contrast, several older physiological trials provided insufficient detail regarding blinding and concealment procedures, although their randomisation processes appeared broadly adequate. One study (10) also raised some concerns because of an inconsistency between the abstract and Methods regarding FiO_2_ allocation, although the trial was otherwise reported as a parallel-group randomised study. These considerations were taken into account in the interpretation of pooled estimates and in the prespecified sensitivity analyses.

### 3.4 Co-primary outcomes

Sample sizes differed slightly across outcomes because pooled denominators were based on the outcome-specific analysable populations reported by the original trials.

### 3.5 Intraoperative hypotension

**Figure 3:**
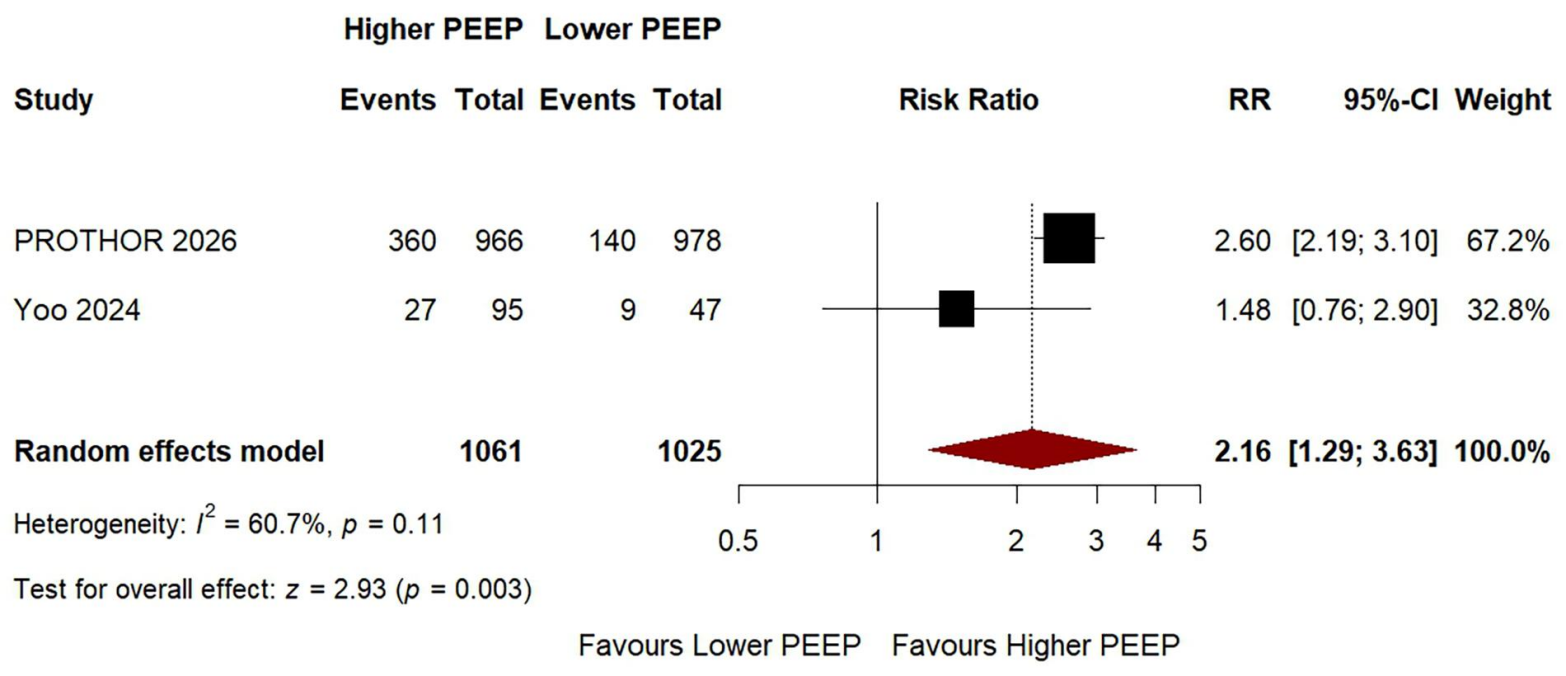
Forest plot of the risk of intraoperative hypotension with higher versus lower PEEP during one-lung ventilation.

Two studies contributed to the meta-analysis of intraoperative hypotension, comprising a total of 2,086 patients. Intraoperative hypotension was defined according to trial-specific criteria. A random-effects model was applied owing to moderate statistical heterogeneity (*I*^2^ = 61%). A higher PEEP strategy was associated with a significantly increased risk of intraoperative hypotension compared with a lower PEEP strategy (RR: 2.16, 95% CI: 1.29–3.63; *p* = 0.003; Figure 3). Both contributing studies showed a numerically higher incidence of hypotension in the higher PEEP group. A separate pooled analysis for clinically significant hypotension was not feasible because reporting was insufficiently consistent across studies. Specifically, only one study (PROTHOR) provided directly extractable data on clinically significant hypotension defined by vasoactive support requirement, precluding formal pooled sensitivity analysis for this endpoint. After exclusion of the PROTHOR trial in the prespecified sensitivity analysis, only one study remained, precluding formal pooling; in that study, a numerically higher incidence of hypotension was observed in the higher PEEP group (28.4% vs. 19.1%), which was not statistically significant (*p* = 0.189).

Data are presented as risk ratio (RR) with 95% confidence interval (CI). Pooled estimate was derived using a random-effects model with restricted maximum likelihood (REML) estimation. Square size is proportional to study weight. The diamond represents the pooled RR and its 95% CI. Higher PEEP was defined as a fixed PEEP level of ≥6 cmH_2_O; lower PEEP was defined as a fixed PEEP level of ≤5 cmH_2_O. For Yoo et al. (9), the higher PEEP arm represents the combined PEEP 6 and PEEP 9 cmH_2_O groups, and the lower PEEP arm represents the PEEP 3 cmH_2_O group. CI, confidence interval; *I*^2^, inconsistency statistic; PEEP, positive end-expiratory pressure; RR, risk ratio.

### 3.6 Postoperative pulmonary complications

**Figure 4:**
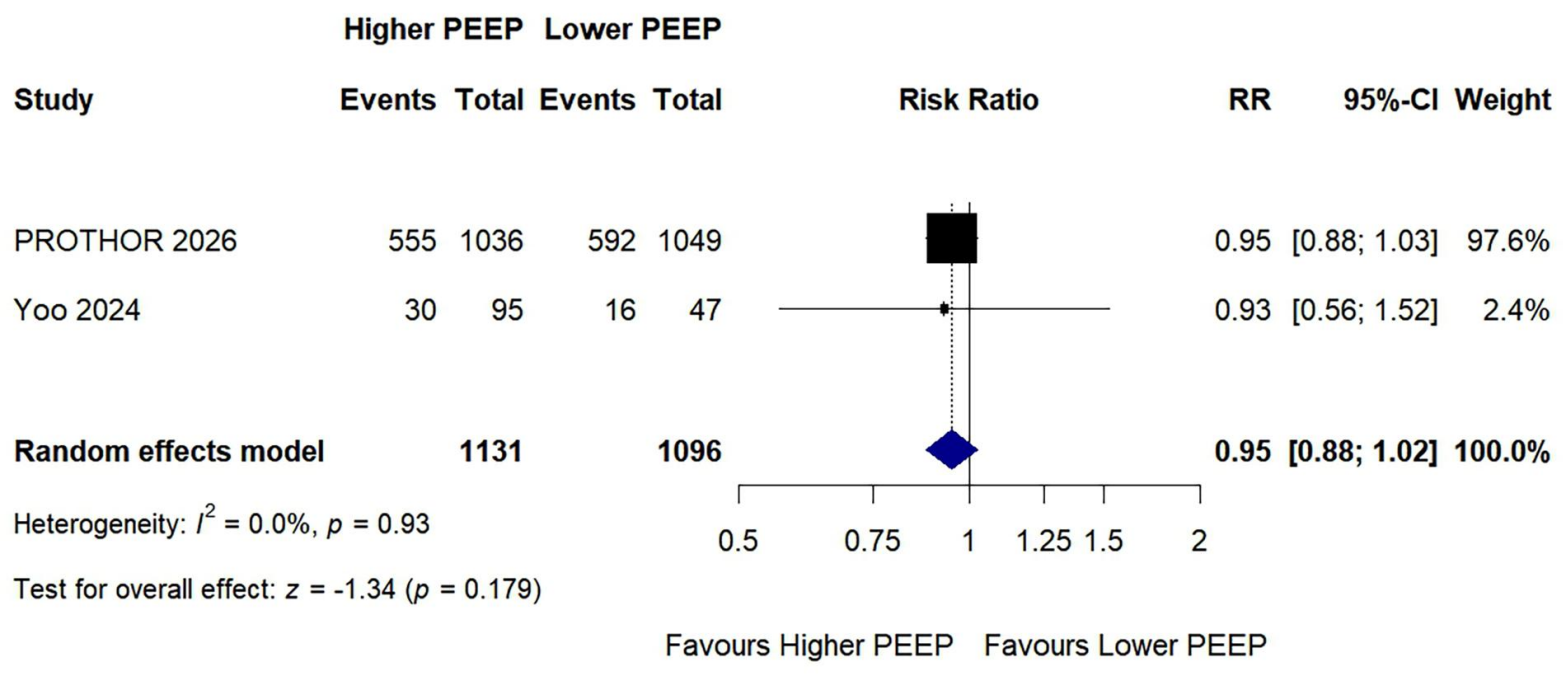
Forest plot of the risk of postoperative pulmonary complications with higher versus lower PEEP during one-lung ventilation.

Two studies contributed to the meta-analysis of postoperative pulmonary complications (PPCs), comprising a total of 2,227 patients. PPCs were analysed using trial-reported composite definitions where available; when an explicit composite was not reported, the prespecified derived composite was used. A higher PEEP strategy did not significantly reduce the risk of PPCs compared with a lower PEEP strategy (RR: 0.95, 95% CI: 0.88–1.02; *p* = 0.179; *I*^2^ = 0%; Figure 4). Statistical heterogeneity was low, and the pooled estimate did not indicate a significant reduction in PPCs with higher PEEP. Restriction to trials reporting an explicit PPC composite did not materially change the conclusion, with no signal of benefit favouring the higher PEEP strategy, although formal pooling remained limited by the small number of contributing studies. After exclusion of the PROTHOR trial, only one study remained, precluding formal pooling; in that study, the direction of effect was similar to that of the primary analysis, with no significant reduction in PPCs in the higher PEEP group (31.6% vs. 34.0%). The certainty of evidence for co-primary and key secondary outcomes, assessed using the GRADE approach, is presented in Table 2.

**Table 2:** GRADE summary of findings for higher vs. lower positive end-expiratory pressure during one-lung ventilation.

Outcome	Studies (N)	Participants	Relative effect (95% CI)	Risk with Lower PEEP	Risk difference with Higher PEEP	Certainty (GRADE)
Co-primary outcomes						
Intraoperative hypotension	2 (2,086)	2,086	RR: 2.16 (1.29–3.63)	143 per 1,000	165 more per 1,000 (41 more to 375 more)	⊕⊕⊕⦻ Moderate^a^
PPCs	2 (2,227)	2,227	RR: 0.95 (0.88–1.02)	564 per 1,000	28 fewer per 1,000 (68 fewer to 11 more)	⊕⊕⊕⦻ Moderate^b^
Key secondary outcomes						
Vasopressor use	2 (2,312)	2,312	RR: 1.05 (0.97–1.13)	514 per 1,000	26 more per 1,000 (15 fewer to 67 more)	⊕⊕⊕⦻ Moderate^b^
New-onset arrhythmia	1 (1,855)	1,855	RR: 2.56 (1.76–3.71)	39 per 1,000	61 more per 1,000 (30 more to 106 more)	⊕⊕⊕⦻ Moderate^c^
Rescue hypoxaemia interventions	1 (1,870)	1,870	RR: 0.37 (0.25–0.56)	88 per 1,000	55 fewer per 1,000 (66 fewer to 39 fewer)	⊕⊕⊕⦻ Moderate^c^
Intraoperative PaO_2_	4 (196)	196	MD: 34.27 mmHg (−11.52 to 80.06)	—	—	⊕⦻⦻⦻ Very low^d^
Peak inspiratory pressure	2 (110)	110	MD: 2.24 cmH_2_O (1.22 to 3.26)	—	—	⊕⊕⊕⦻ Moderate^e^
Dynamic compliance	1 (48)	48	Not pooled	—	—	⊕⊕⦻⦻ Low^f^

CI, confidence interval; GRADE, Grading of Recommendations Assessment, Development and Evaluation; MD, mean difference; PEEP, positive end-expiratory pressure; PPCs, postoperative pulmonary complications; RR, risk ratio. ⊕⊕⊕⊕ High certainty; ⊕⊕⊕⦻ Moderate certainty; ⊕⊕⦻⦻ Low certainty; ⊕⦻⦻⦻ Very low certainty.

Risk estimates for dichotomous outcomes are expressed as risk ratio (RR) with 95% confidence interval (CI). Effect estimates for continuous outcomes are expressed as mean difference (MD) with 95% CI. Absolute risk differences for dichotomous outcomes were calculated based on the baseline risk observed in the lower PEEP group. Certainty of evidence was assessed using the GRADE (Grading of Recommendations Assessment, Development and Evaluation) approach. For single-study outcomes (new-onset arrhythmia, rescue hypoxaemia interventions), results reflect the findings of the PROTHOR trial only and were not independently replicated. Dynamic compliance was available from a single study (24) and was not pooled.

a Downgraded one level for inconsistency (*I*² = 60.7%).

b Downgraded one level for imprecision (95% CI includes the null value).

c Downgraded one level for imprecision (single study; results not independently replicated).

d Downgraded three levels: risk of bias (several contributing studies rated as some concerns), inconsistency (*I*² = 94.2%), and indirectness (heterogeneous patient positioning, FiO_2_ settings, and surgical populations across trials).

e Downgraded one level for risk of bias (contributing studies rated as some concerns regarding blinding of outcome assessors).

f Downgraded two levels for risk of bias and imprecision (single study with small sample size).

Data are presented as risk ratio (RR) with 95% confidence interval (CI). Pooled estimate was derived using a random-effects model with restricted maximum likelihood (REML) estimation. Square size is proportional to study weight. The diamond represents the pooled RR and its 95% CI. Higher PEEP was defined as a fixed PEEP level of ≥6 cmH_2_O; lower PEEP was defined as a fixed PEEP level of ≤5 cmH_2_O. For Yoo et al. (9), the higher PEEP arm represents the combined PEEP 6 and PEEP 9 cmH_2_O groups, and the lower PEEP arm represents the PEEP 3 cmH_2_O group. PPCs were defined according to trial-specific composite definitions; where no explicit composite was reported, a derived composite was constructed from available individual components. CI, confidence interval; *I*^2^, inconsistency statistic; PEEP, positive end-expiratory pressure; PPCs, postoperative pulmonary complications; RR, risk ratio.

### 3.7 Secondary outcomes

#### 3.7.1 Haemodynamic and intraoperative safety outcomes

Data on intraoperative vasopressor use were available from two studies using comparable definitions (any vasopressor use). Pooled analysis demonstrated no statistically significant difference between higher and lower PEEP groups (RR: 1.05, 95% CI: 0.97–1.13; *p* = 0.212; *I*^2^ = 0%). In the PROTHOR trial, any vasopressor use was reported in 54.4% of the higher PEEP group vs. 51.4% of the lower PEEP group. Extractable data on new-onset intraoperative arrhythmia were available largely from the PROTHOR trial, in which arrhythmia occurred more frequently in the higher PEEP group than in the lower PEEP group (RR: 2.56, 95% CI: 1.76–3.71; 9.9% vs. 3.9%). Extractable data on rescue hypoxaemia interventions were also available largely from the PROTHOR trial, in which rescue manoeuvres were less frequently required in the higher PEEP group than in the lower PEEP group (RR: 0.37, 95% CI: 0.25–0.56; 3.3% vs. 8.8%).

#### 3.7.2 Oxygenation and respiratory mechanics outcomes

Although the PaO_2_/FiO_2_ ratio was prespecified as the preferred oxygenation metric, PaO_2_ was the most consistently extractable continuous oxygenation variable across the included trials and was therefore used for quantitative synthesis. Four studies contributed extractable data for intraoperative PaO_2_. Using measurements obtained at the prespecified or closest comparable time point during OLV, the pooled estimate did not demonstrate a statistically significant difference between higher and lower PEEP strategies (MD: 34.27 mmHg, 95% CI: −11.52 to 80.06; *p* = 0.142; *I*^2^ = 94.2%; Supplementary Figure 1). The 95% prediction interval was −205.79 to 273.70 mmHg, confirming that the true effect of higher vs. lower PEEP on intraoperative oxygenation is highly variable across settings and populations, and that the pooled estimate should not be used to guide clinical decisions. Prediction intervals could not be calculated for analyses based on two studies (intraoperative hypotension, PPCs, and PIP), as the t-distribution required for this calculation is undefined at zero degrees of freedom. Statistical heterogeneity was substantial, likely reflecting differences in patient positioning, FiO_2_ settings, PEEP contrasts, and surgical populations across trials. Notably, one study (23) reported a numerically lower PaO_2_ in the higher PEEP group, a finding observed in the trial conducted in the supine position for anterior mediastinal surgery, in contrast to the lateral decubitus position used in all other included studies. Although several studies recorded the PaO_2_/FiO_2_ ratio, extractable continuous data suitable for analysis were primarily available from two studies (9, 24) during or shortly after OLV; both studies showed higher PaO_2_/FiO_2_ ratios in the higher PEEP groups. Extractable data on dynamic compliance were available from a single study (24), in which higher PEEP was associated with greater dynamic compliance during OLV (38.29 ± 7.27 vs. 30.46 ± 7.39 mL/cmH_2_O at 30 min of OLV); formal pooling was not performed owing to the availability of data from a single study. Two studies (23, 24) contributed continuous data on peak inspiratory pressure (PIP) during OLV, and the pooled analysis demonstrated a small but statistically significant increase in PIP associated with the use of higher PEEP (MD 2.24 cmH_2_O, 95% CI: 1.22–3.26; *p* < 0.001; *I*^2^ = 0%; Supplementary Figure 2). Additional physiological markers were sparsely reported. Choi et al. (23) reported no significant between-group differences in the intrapulmonary shunt fraction (Qs/Qt) when comparing fixed PEEP with the control group. In a single small trial evaluating a dose-response relationship, Leong et al. (7) reported that the physiologic dead space to tidal volume ratio (Vd/Vt) was lowest at a PEEP level of 8 cmH_2_O compared with 0, 5, or 10 cmH_2_O.

#### 3.7.3 Postoperative clinical and resource-use outcomes

Postoperative resource utilisation, including unexpected ICU admissions and hospital length of stay, was reported mainly in the PROTHOR trial and, to a lesser extent, in Yoo et al. (9). In the PROTHOR trial, unexpected postoperative ICU admissions were similar between groups, and no clear between-group differences in hospital length of stay were reported. Mortality was reported primarily in the PROTHOR trial and was low in both groups, with no significant between-group difference.

### 3.8 Subgroup, sensitivity, and publication bias analyses

#### 3.8.1 Prespecified subgroup analyses

Although subgroup analyses were prespecified based on surgery type, surgical approach, PEEP contrast magnitude, and the use of recruitment manoeuvres, the limited number of studies contributing to each quantitative synthesis precluded formal statistical pooling. A narrative evaluation of the study characteristics indicated that clinical outcomes (such as PPCs and intraoperative hypotension) were reported almost exclusively by one large multicentre trial (PROTHOR) and one medium-sized single-centre trial (9). Conversely, smaller single-centre trials primarily contributed to the physiological outcomes. Consequently, meaningful statistical testing for subgroup interactions was not feasible.

#### 3.8.2 Sensitivity analyses

Prespecified sensitivity analyses were performed to test the stability of the primary findings. After exclusion of the large PROTHOR trial, only one study (9) remained for the co-primary outcomes of PPCs and intraoperative hypotension, leaving only narrative comparison possible. As reported in the primary analysis section, the direction of effect in this remaining study was similar to that of the primary analysis. Because data on new-onset arrhythmia and rescue hypoxaemia interventions were exclusively derived from the PROTHOR trial, this sensitivity analysis could not be applied to those outcomes. Continuous physiological outcomes, such as PaO_2_ and dynamic compliance, were unaffected by this sensitivity analysis, as the PROTHOR trial did not contribute extractable continuous data to these specific syntheses.

The prespecified sensitivity analysis excluding studies at overall high risk of bias was not applicable because no included study was judged to be at overall high risk of bias. In an additional sensitivity analysis restricted to studies judged to be at low risk of bias [PROTHOR (13), Yoo et al. (9), and Choi et al. (23)], the pooled estimates for the co-primary outcomes of PPCs and intraoperative hypotension remained unchanged, because both contributing studies were at low risk of bias. For continuous physiological outcomes, restricting the analysis to low-risk studies left only a single study (23) for variables such as PaO_2_ and dynamic compliance, leaving only descriptive interpretation possible.

Alternative pairwise comparisons were conducted for the included multi-arm trials [e.g., comparing 9 cmH_2_O vs. 3 cmH_2_O in Yoo et al. (9), or 10 cmH_2_O vs. 0 cmH_2_O in (7, 10)] instead of the merged higher vs. lower PEEP groups used in the primary analysis. These alternative comparisons yielded point estimates and directions of effect that were consistent with the primary merged analyses. Use of alternative intraoperative time points for continuous outcomes (e.g., measurements at 60 min of OLV instead of the primary 20–30 min time point) did not materially alter the pooled physiological results. When the analysis of intraoperative PaO_2_ was restricted to studies conducted in the lateral decubitus position [excluding (23)], a statistically significant benefit of higher PEEP was observed (MD: 49.26 mmHg, 95% CI: 1.72–96.81; *p* = 0.042; *I*^2^ = 94.5%), although substantial heterogeneity persisted and the result should be interpreted with caution.

#### 3.8.3 Publication bias and small-study effects

Formal assessment of publication bias and small-study effects, such as the visual inspection of funnel plots or Egger's test, was not undertaken because fewer than 10 studies contributed to each quantitative synthesis.

## 4 Discussion

This systematic review and meta-analysis of eight randomised controlled trials comparing higher vs. lower fixed PEEP strategies during one-lung ventilation for thoracic surgery yielded two central findings. First, a higher fixed PEEP strategy was associated with a significantly increased risk of intraoperative hypotension (RR: 2.16, 95% CI: 1.29–3.63). Second, higher PEEP did not significantly reduce the risk of postoperative pulmonary complications (RR: 0.95, 95% CI: 0.88–1.02). Although some smaller physiological trials suggested directionally favourable effects on intraoperative oxygenation, and single-study data suggested improved dynamic compliance, these signals did not translate into measurable postoperative clinical benefit. Taken together, these findings indicate a clear dissociation between short-term physiological improvement and clinically meaningful patient outcomes, and suggest that the haemodynamic cost of higher fixed PEEP is not balanced by a measurable improvement in clinically meaningful postoperative outcomes.

The finding that higher PEEP more than doubled the risk of intraoperative hypotension is clinically important and mechanistically plausible. Elevated intrathoracic pressure reduces venous return to the right heart and increases right ventricular afterload through compression of pulmonary capillaries; mechanistically, these changes would be expected to reduce cardiac output and predispose to systemic hypotension (25). In the PROTHOR trial, intraoperative hypotension occurred in 37.3% of patients in the higher PEEP group compared with 14.3% in the lower PEEP group, representing an absolute risk increase of approximately 23 percentage points (13). The pooled estimate in our analysis (RR: 2.16) is consistent with this trial-level finding, and the sensitivity analysis excluding PROTHOR showed a directionally similar effect in the remaining study. The concurrent increase in new-onset arrhythmia observed in the PROTHOR trial (RR: 2.56, 95% CI: 1.76–3.71; 9.9% vs. 3.9%) further supports the interpretation that higher PEEP imposes a substantive haemodynamic burden in this surgical population (13). These findings carry direct clinical implications. In patients at elevated haemodynamic risk — including those with reduced cardiac reserve, pre-existing hypertension, or anticipated prolonged one-lung ventilation — the routine application of higher fixed PEEP should be accompanied by heightened intraoperative haemodynamic monitoring, including continuous arterial pressure monitoring and preparedness for prompt vasoactive intervention. Anaesthesiologists should explicitly weigh the haemodynamic cost of higher fixed PEEP before adopting this strategy on a routine basis.

The absence of a significant reduction in PPCs with higher PEEP (RR: 0.95, 95% CI: 0.88–1.02) is consistent with the primary result of the PROTHOR trial and extends that finding to the broader evidence base (13). Importantly, the point estimate was close to unity, arguing against a clinically important protective effect that was merely missed because of limited precision. Several mechanisms may explain why higher PEEP failed to translate into fewer PPCs. Intraoperative atelectasis during OLV is driven not only by loss of end-expiratory lung volume but also by absorbed atelectasis in the dependent lung and impaired hypoxic pulmonary vasoconstriction; PEEP addresses only one component of this multifactorial process (1, 26–28). Furthermore, the haemodynamic compromise associated with higher PEEP could plausibly offset any pulmonary protective effect by reducing perfusion pressure and impairing oxygen delivery to vulnerable lung tissue, although the included trials were not designed to test this mechanistic pathway directly. Our findings also highlight a broader methodological problem in thoracic anaesthesia research: transient intraoperative physiological improvements should not be assumed to predict downstream postoperative benefit. Prior small single-centre trials frequently interpreted improvements in surrogate physiological endpoints, such as intraoperative PaO_2_ or dynamic compliance, as evidence of lung protection. The present analysis suggests that this inference may be misplaced. This surrogate endpoint paradox should serve as a methodological caution for future perioperative ventilation research, in which transient physiological improvement should not be assumed to predict downstream clinical benefit.

The pooled analysis of intraoperative PaO_2_ across four studies was directionally favourable but statistically inconclusive, and extreme heterogeneity (*I*^2^ = 94.2%) substantially limits confidence in the pooled estimate. The divergent direction of effect in Choi etal. (23) is consistent with known differences in pulmonary physiology between supine and lateral decubitus positioning, where the gravitational distribution of blood flow and the pattern of atelectasis formation differ substantially. When the analysis was restricted to studies conducted in the lateral decubitus position, the direction of effect became more favourable after exclusion of Choi et al. (23), although substantial heterogeneity remained, likely driven by differences in FiO_2_ settings and PEEP contrasts across trials. Dynamic compliance data were available from a single study only and cannot be pooled; the improvement observed in Kim et al. (24) is physiologically plausible, as PEEP may increase functional residual capacity, reduce cyclic alveolar collapse, and improve recruitment in susceptible lung units (1). The small but statistically significant increase in peak inspiratory pressure with higher PEEP (MD: 2.24 cmH_2_O, 95% CI: 1.22–3.26; *I*^2^ = 0%) reflects the expected mechanical consequence of applying higher airway pressure. This finding also reminds clinicians that higher PEEP is not without respiratory mechanical cost, even when tidal volumes are kept constant.

Our findings are consistent with the growing body of evidence from general surgical populations suggesting that higher PEEP does not universally reduce pulmonary complications. In mechanically ventilated patients undergoing major abdominal surgery, the PROVHILO trial similarly demonstrated that higher PEEP with recruitment manoeuvres did not reduce PPCs compared with lower PEEP, while increasing the risk of intraoperative hypotension (29). In obese patients undergoing a mixed population of abdominal and non-abdominal surgical procedures — a population with markedly elevated baseline atelectasis risk and distinct respiratory mechanics compared with non-obese thoracic surgical patients — the PROBESE trial similarly demonstrated that intraoperative high PEEP (12 cmH_2_O) with recruitment manoeuvres did not reduce PPCs compared with low PEEP (4 cmH_2_O), despite improvements in intraoperative oxygenation (28). A subsequent patient-level meta-analysis of PROVHILO, iPROVE, and PROBESE further confirmed that intraoperative PEEP level was not independently associated with postoperative pulmonary complications across these three large trials, reinforcing the conclusion that the absence of clinical benefit from higher fixed PEEP is a robust finding that transcends specific surgical populations, body positions, and levels of abdominal pressure (29). This parallel further supports the notion that the haemodynamic cost of higher PEEP may reflect a broader physiological trade-off that is not unique to thoracic surgery (30). Current recommendations for lung-protective ventilation during OLV generally support low tidal volume ventilation with moderate or individualised PEEP, while acknowledging that the optimal PEEP target remains uncertain (31–34). The results of our analysis, anchored by the PROTHOR trial, provide the most robust evidence to date that higher fixed PEEP (≥8–10 cmH_2_O) during OLV does not improve clinical outcomes and significantly increases haemodynamic risk, findings that should inform future guideline updates.

Several limitations of this review warrant consideration. First, the number of studies contributing to each quantitative synthesis was small; for the co-primary outcomes, only two studies were poolable, and the pooled estimates were heavily influenced by the PROTHOR trial. This concentration of evidence limits our ability to formally explore heterogeneity through subgroup analyses and precludes assessment of publication bias. However, the dominance of PROTHOR reflects the availability of a large, high-quality, low-risk-of-bias anchor trial that meets the optimal information size for both co-primary outcomes, thereby reducing the risk of random error and correcting the systematic overestimation of benefit that characterised earlier smaller trials. Second, the physiological outcomes were derived predominantly from small single-centre trials with heterogeneous PEEP contrasts, FiO_2_ settings, surgical populations, and patient positioning, resulting in extreme statistical heterogeneity that limits the reliability of pooled estimates for these endpoints. Third, several outcome definitions—particularly for hypotension, PPC composites, and rescue intraoperative interventions—were not fully standardised across trials, which may have introduced clinical heterogeneity despite our efforts to preserve trial-level definitions. Finally, one included trial (23) was conducted in the supine position for anterior mediastinal surgery, representing a distinct physiological context from the lateral decubitus positioning used in all other trials, although this issue was partly explored in sensitivity analysis. Additionally, formal meta-regression to explore the potential influence of tidal volume, surgery type, and patient body position on the pooled estimates was not feasible given the small number of studies contributing to each quantitative synthesis; such analyses are typically recommended only when a minimum of approximately 10 studies are available per covariate, a threshold not met in the current review.

In conclusion, this systematic review and meta-analysis demonstrates that higher fixed PEEP during one-lung ventilation for thoracic surgery significantly increases the risk of intraoperative hypotension without reducing the incidence of postoperative pulmonary complications. Short-term physiological improvements in oxygenation and respiratory mechanics do not translate into clinical benefit, underscoring the hazard of relying on surrogate endpoints to guide ventilation strategy. The routine use of higher fixed PEEP during OLV should therefore be approached with caution, and clinical decisions should explicitly account for the haemodynamic cost of this strategy. These findings should not be interpreted as evidence against the use of PEEP *per se*, but rather against the routine application of higher fixed PEEP levels without individualisation. Future research should move beyond the question of optimal fixed PEEP levels and instead evaluate whether individualised PEEP titration—guided by driving pressure optimisation or electrical impedance tomography—can preserve the physiological benefits of higher PEEP while avoiding haemodynamic compromise. Emerging evidence suggests that such an approach may be promising: the iPROVE-OLV trial demonstrated that a perioperative individualised open-lung approach, incorporating EIT-guided PEEP titration, reduced severe postoperative pulmonary complications compared with standard lung-protective ventilation, without the haemodynamic penalties observed with fixed higher PEEP strategies (31). Future trials should prospectively evaluate whether such individualised approaches can achieve true lung protection without clinical harm in the thoracic surgical population (26, 35).

## Data Availability

The original contributions presented in the study are included in the article/Supplementary Material, further inquiries can be directed to the corresponding author.
