# Supplementary material for: Higher vs. lower positive end-expiratory pressure during one-lung ventilation for thoracic surgery: a systematic review and meta-analysis
Source: Front Surg. 2026 May 11;13:1838287. doi: 10.3389/fsurg.2026.1838287 (PMC13199356; doi:10.3389/fsurg.2026.1838287)
Supplement: Supplementary file 1 [file Datasheet1.pdf]

Supplementary Figure 1. Forest plot of intraoperative PaO_2 with higher versus lower PEEP during one-lung ventilation.

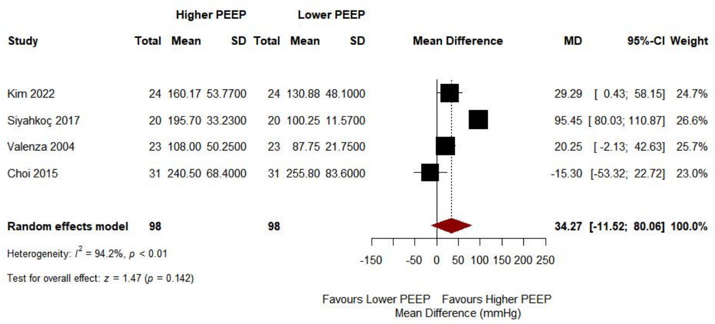

Data are presented as mean difference (MD) with 95% confidence interval (CI). Pooled estimate was derived using a random-effects model with restricted maximum likelihood (REML) estimation. Square size is proportional to study weight. The diamond represents the pooled MD and its 95% CI. Higher PEEP was defined as a fixed PEEP level of ≥6 cmH_2O; lower PEEP was defined as a fixed PEEP level of ≤5 cmH_2O. For Valenza 2004, PaO_2 values originally reported in kPa were converted to mmHg (×7.5) prior to analysis; data represent the overall patient population at the lateral decubitus plus treatment time point. Choi 2015 was conducted in the supine position for anterior mediastinal surgery, in contrast to the lateral decubitus position used in all other included studies, which may account for the divergent direction of effect observed in that trial. Substantial heterogeneity was present across studies (I^2 = 94.2%), and the pooled estimate should be interpreted with caution.

CI, confidence interval; I^2, inconsistency statistic; MD, mean difference; OLV, one-lung ventilation; PEEP, positive end-expiratory pressure.

Supplementary Figure 2. Forest plot of peak inspiratory pressure with higher versus lower PEEP during one-lung ventilation.

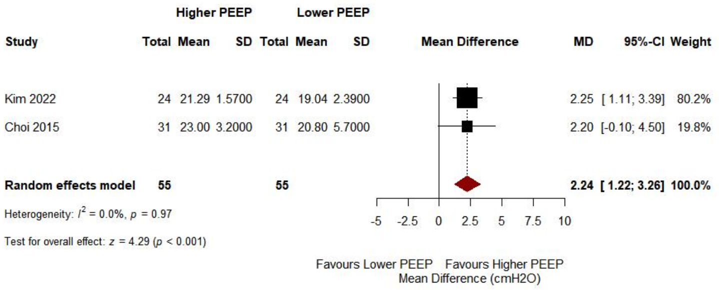

Data are presented as mean difference (MD) with 95% confidence interval (CI). Pooled estimate was derived using a random-effects model with restricted maximum likelihood (REML) estimation. Square size is proportional to study weight. The diamond represents the pooled MD and its 95% CI. Higher PEEP was defined as a fixed PEEP level of ≥6 cmH_2O; lower PEEP was defined as a fixed PEEP level of ≤5 cmH_2O. Data from Kim 2022 represent measurements at 30 minutes of one-lung ventilation; data from Choi 2015 represent measurements at OLV30. For Choi 2015, only the control and fixed-PEEP arms were included; the alveolar recruitment arm was excluded from the analysis.

CI, confidence interval; I^2, inconsistency statistic; MD, mean difference; OLV, one-lung ventilation; PEEP, positive end-expiratory pressure; PIP, peak inspiratory pressure.

Supplementary Appendix 1

Full Electronic Search Strategies

The following search strategies were used to identify eligible randomised controlled trials. The final search was run on 7 March 2026. All searches were conducted without language or date restrictions.

1. MEDLINE (via PubMed)

Database: PubMed

Date searched: 7 March 2026

Search string:

```
("one-lung ventilation"[Mesh] OR "one lung ventilation"[tiab] OR "single lung ventilation"[tiab] OR OLV[tiab])
```

AND

```
("positive end-expiratory pressure"[Mesh] OR PEEP[tiab] OR "recruitment maneuver"[tiab] OR "recruitment manoeuvre"[tiab] OR "alveolar recruitment"[tiab])
```

AND

```
("thoracic surgery"[Mesh] OR "lung resection"[tiab] OR lobectomy[tiab] OR pneumonectomy[tiab] OR esophagectomy[tiab] OR oesophagectomy[tiab] OR VATS[tiab] OR "video-assisted thorac*"[tiab])
```

AND

```
("randomized controlled trial"[pt] OR randomized[tiab] OR randomised[tiab] OR randomly[tiab])
```

2. Embase (via Embase.com)

Database: Embase

Date searched: 7 March 2026

Search string:

```
('one lung ventilation'/exp OR 'one lung ventilation':ti,ab,kw OR 'single lung ventilation':ti,ab,kw OR OLV:ti,ab,kw)
```

AND

```
('positive end expiratory pressure'/exp OR PEEP:ti,ab,kw OR 'recruitment maneuver':ti,ab,kw OR 'recruitment manoeuvre':ti,ab,kw OR 'alveolar recruitment':ti,ab,kw)
```

AND

```
('thoracic surgery'/exp OR 'lung resection':ti,ab,kw OR lobectomy:ti,ab,kw OR pneumonectomy:ti,ab,kw OR esophagectomy:ti,ab,kw OR oesophagectomy:ti,ab,kw OR VATS:ti,ab,kw OR 'video-assisted thorac*':ti,ab,kw)
```

AND

```
('randomized controlled trial'/exp OR randomized:ti,ab,kw OR randomised:ti,ab,kw OR randomly:ti,ab,kw)
```

3. Cochrane Central Register of Controlled Trials (CENTRAL)

Database: CENTRAL (Cochrane Library)

Date searched: 7 March 2026

Search string:

```
("one lung ventilation" OR "single lung ventilation" OR OLV)
```

AND

```
("positive end-expiratory pressure" OR PEEP OR "recruitment maneuver" OR "recruitment manoeuvre" OR "alveolar recruitment")
```

AND

```
("thoracic surgery" OR "lung resection" OR lobectomy OR pneumonectomy OR esophagectomy OR oesophagectomy OR VATS OR "video-assisted thoracic")
```

4. ClinicalTrials.gov

Database: ClinicalTrials.gov (https://clinicaltrials.gov)

Date searched: 7 March 2026

Search string:

```
("one lung ventilation" OR OLV OR "single lung ventilation")
```

AND

```
(PEEP OR "positive end-expiratory pressure" OR "recruitment maneuver" OR "recruitment manoeuvre")
```

AND

```
("thoracic surgery" OR lobectomy OR pneumonectomy OR esophagectomy OR oesophagectomy OR VATS)
```

5. WHO International Clinical Trials Registry Platform (WHO ICTRP)

Database: WHO ICTRP (https://trialsearch.who.int)

Date searched: 7 March 2026

Search string:

```
("one lung ventilation" OR OLV OR "single lung ventilation")
```

AND

```
(PEEP OR "positive end-expiratory pressure" OR "recruitment maneuver" OR "recruitment manoeuvre")
```

AND

```
("thoracic surgery" OR lobectomy OR pneumonectomy OR esophagectomy OR oesophagectomy OR VATS)
```

Note: Search strategies for ClinicalTrials.gov and WHO ICTRP were used to identify ongoing or unpublished trials. No randomised trial filter was applied to these two registries, as randomisation status is not consistently indexed. The CENTRAL search did not require an additional randomised trial filter, as CENTRAL indexes only controlled trials by design.
